# An optimized workflow to improve reliability of detection of *KIAA1549:BRAF* fusions from RNA sequencing data

**DOI:** 10.1007/s00401-020-02167-1

**Published:** 2020-05-31

**Authors:** Alexander C. Sommerkamp, Sebastian Uhrig, Damian Stichel, Pascal St-Onge, Pengbo Sun, Natalie Jäger, Andreas von Deimling, Felix Sahm, Stefan M. Pfister, Andrey Korshunov, Daniel Sinnett, Nada Jabado, Annika K. Wefers, David T. W. Jones

**Affiliations:** 1 Hopp Children’s Cancer Center Heidelberg (KiTZ), Heidelberg, Germany; 2 Pediatric Glioma Research Group, German Cancer Consortium (DKTK), German Cancer Research Center (DKFZ), Heidelberg, Germany; 3 Faculty of Biosciences, Heidelberg University, Heidelberg, Germany; 4 Computational Oncology Group, Molecular Diagnostics Program at the National Center for Tumor Diseases (NCT) and DKFZ, Heidelberg, Germany; 5 Division of Applied Bioinformatics, German Cancer Research Center (DKFZ), Heidelberg, Germany; 6 Department of Neuropathology, University Hospital Heidelberg, Heidelberg, Germany; 7 Clinical Cooperation Unit Neuropathology, German Cancer Consortium (DKTK), German Cancer Research Center (DKFZ), Heidelberg, Germany; 8 Division of Hematology-Oncology, Charles-Bruneau Cancer Centre, CHU Sainte-Justine, Montreal, Canada; 9 Division of Pediatric Neurooncology, German Cancer Consortium (DKTK), German Cancer Research Center (DKFZ), Heidelberg, Germany; 10 Department of Pediatric Oncology, Hematology and Immunology, University Hospital Heidelberg, Heidelberg, Germany; 11 Department of Pediatrics, Faculty of Medicine, University of Montreal, Montreal, QC Canada; 12 Department of Pediatrics, Faculty of Medicine, McGill University, Montreal, Canada

The *KIAA1549:BRAF* fusion is the most common alteration in pilocytic astrocytoma (PA). It is generated by a focal tandem duplication at 7q34 and acts as an oncogene by driving the mitogen-activated protein kinase (MAPK) pathway [4]. Detection of this characteristic genetic event is of high clinical relevance, both for its diagnostic/prognostic relevance and as a therapeutic target. RNA sequencing (RNA-Seq) of fresh-frozen or formalin-fixed paraffin-embedded (FFPE) tissue has recently gained popularity in the diagnostic setting [1]. By identifying split reads that map to two different genomic loci, RNA-Seq data can be used to detect expressed fusion genes. Several tools have been developed for this purpose, including Arriba (https://github.com/suhrig/arriba), FusionCatcher (https://github.com/ndaniel/fusioncatcher) and STAR-Fusion (https://github.com/STAR-Fusion/STAR-Fusion). Previous studies have suggested that the *KIAA1549:BRAF* fusion is expressed at a low level [5–7], but the reliability of detection of this important fusion using different RNA-Seq analysis pipelines has not been examined so far.

To this end, we generated RNA-Seq data (polyA-enriched, TruSeq Stranded, 2 × 100 bp paired-end reads) from 22 fresh-frozen pediatric PA tumor samples, in which a *KIAA1549:BRAF* fusion had previously been identified by whole-genome sequencing (WGS) [3]. The raw data was subsequently aligned by STAR [2] (v2.7.3a), and gene fusions were identified using Arriba (v1.1.0). Despite a total read count of about 200 million reads per sample (Fig. 1a), the *KIAA1549:BRAF* fusion was only correctly identified in 14/22 samples (Fig. 1b). In three additional samples, Arriba had identified but then discarded the fusion, as it was supported by just one sequencing read. In five samples, the fusion was not detected at all with this workflow. To investigate the influence of sequencing depth, we re-sequenced these five samples, substantially increasing their total read count to more than 500 million reads per sample (Fig. 1a). Surprisingly, however, we were still unable to detect the fusion in four of these five samples, only slightly changing the overall result (Fig. 1b). The detection rate was not significantly different between samples with a *KIAA1549* exon 16—*BRAF* exon 9 (16:9) or with the 15:9 fusion variant (Online Resource Fig. 1a).

**Fig. 1:**
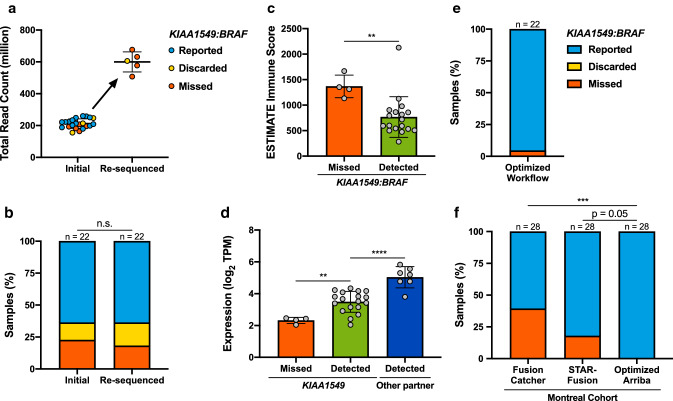
**a** Total read count of 22 fresh-frozen pediatric PA tumor samples sequenced by RNA-Seq. The five samples in which the *KIAA1549:BRAF* fusion was missed were re-sequenced, increasing their total read number and allowing detection of the fusion in one additional sample (although still in the ‘discarded’ list). Mean ± SD. **b** Relative frequency of reported, discarded and missed *KIAA1549:BRAF* fusions in the initial RNA-Seq data and after re-sequencing. Chi-square test on the underlying absolute values. **c** Immune score calculated by ESTIMATE as an indicator of immune cell content in samples with a missed or detected *KIAA1549:BRAF* fusion. Mean ± SD. Unpaired *t* test. **d** Expression of *KIAA1549* in samples with a missed or detected *KIAA1549:BRAF* fusion as well as expression of the upstream fusion partner in alternative fusion variants (see main text). Mean ± SD. One-way ANOVA followed by Tukey multiple comparisons test. **e** Relative frequency of reported, discarded and missed *KIAA1549:BRAF* fusions after workflow optimization. **f** Relative frequency of reported and missed *KIAA1549:BRAF* fusions in an independent diagnostic cohort in comparison to FusionCatcher and STAR-Fusion as the previous standard analysis tools. Fisher's exact test on the underlying absolute values. For all panels: *p < 0.05, **p < 0.01, ***p < 0.001, ****p < 0.0001, n.s.: not significant

Next, we investigated different factors that could influence the detectability. The immune cell content, evaluated by ESTIMATE [8], was significantly lower in those samples in which the fusion was detected (reported or discarded) compared to those in which the fusion was missed (Fig. 1c), this suggests that a higher tumor cell content facilitates fusion detection. The amplitude of the genomic 7q34 gain as a further measure of tumor purity pointed in a similar direction (Online Resource Fig. 1b and 2). The expression level of the fusion partner genes, *KIAA1549* (Fig. 1d) and *BRAF* (Online Resource Fig. 1c), was also significantly higher in cases where the fusion was detected. Interestingly, *BRAF* fusions with alternative fusion partners (*FAM131B*, *GNAI1*, *MKRN1* or *RNF130*) were detected without problems, and the expression of these alternative 5′ genes was consistently higher than that of *KIAA1549* (Fig. 1d). The estimated library size (a measure of the complexity captured by the RNA-Seq library) showed a trend towards correlating with detectability (Online Resource Fig. 1d), but had levels in both groups that were above those typically considered to cause general problems in fusion detection (< 30 million; authors’ unpublished observations). Furthermore, we could not exclude an influence of the library preparation protocol. Fusion analysis of an older RNA-Seq cohort was significantly more sensitive compared to the cohort presented here (Online Resource Fig. 1e), with the only obvious difference being the library preparation protocol (ribosome-depleted total RNA vs. polyA capture). Likely, a combination of all of these factors determines the overall detectability for a given sample. In particular, however, the samples in which the *KIAA1549:BRAF* fusion was missed ranked significantly worse for *KIAA1549* expression and tumor cell content (Online Resource Fig. 1f–g).

Analyzing the data using FusionCatcher (v1.20) did not improve the overall result (Online Resource Table 1). FusionCatcher missed some fusions that were detected by Arriba but also reported one that was missed by Arriba. Therefore, we hypothesized that the raw sequencing data might contain fusion-relevant information that is differently processed by the algorithms. Indeed, scanning the raw FASTQ files for sequences spanning the breakpoint of *KIAA1549* and *BRAF* (16:9 and 15:9) using the UNIX utility *grep* revealed matching reads in all samples. Further analysis showed that these split reads were not always properly aligned by STAR, which has known issues with overlapping paired-end reads and split reads with a short overhang, and were thus not visible to downstream processing by Arriba.

To overcome these limitations, we tested different parameters that have recently been incorporated into STAR. We found the settings *–peOverlapNbasesMin 10* and *–alignSplicedMateMapLminOverLmate 0.5* to improve the alignment of split reads from our paired-end sequencing data. In addition, we developed a new version of Arriba (v1.2.0) that is able to detect fusions with only one supporting read if they are included in a curated list of known fusions. This should reduce the number of false negatives observed with earlier versions of Arriba. These modifications substantially improved overall detection of the *KIAA1549:BRAF* fusion (Fig. 1e) and increased the confidence of identified fusions (Online Resource Fig. 1h). We further validated this optimized workflow in an independent diagnostic cohort, and found it to significantly outperform the previous standard analysis tools FusionCatcher and STAR-Fusion (Fig. 1f).

Finally, we analyzed RNA-Seq data from a set of > 1000 FFPE tissue samples processed in a diagnostic setting [6]. Importantly, the more sensitive detection parameters did not result in any false positive calls in non-*KIAA1549:BRAF* PA or other tumor types (100% specificity).

The presented modifications to STAR and Arriba considerably improved the detection rate of *KIAA1549:BRAF* fusions from RNA-Seq data in research and diagnostic settings. We expect that these improvements are likely to also result in increased fusion detection sensitivity in other contexts. It should be noted, however, that not all fusion-supporting evidence contained in the raw read data was picked up by our approach, even after optimization. Therefore, additional enhancements of STAR, Arriba and related tools will be needed in order to further improve the detection rate.

## Electronic supplementary material

Below is the link to the electronic supplementary material.

Supplementary file1 (PDF 157 kb)
