# Supplementary material for: An optimized workflow to improve reliability of detection of KIAA1549:BRAF fusions from RNA sequencing data
Source: Acta Neuropathol. 2020 May 31;140(2):237–9. doi: 10.1007/s00401-020-02167-1 (PMC7360662; doi:10.1007/s00401-020-02167-1)
Supplement: Supplementary file 1 — Supplementary file1 (PDF 157 kb) [file 401_2020_2167_MOESM1_ESM.pdf]

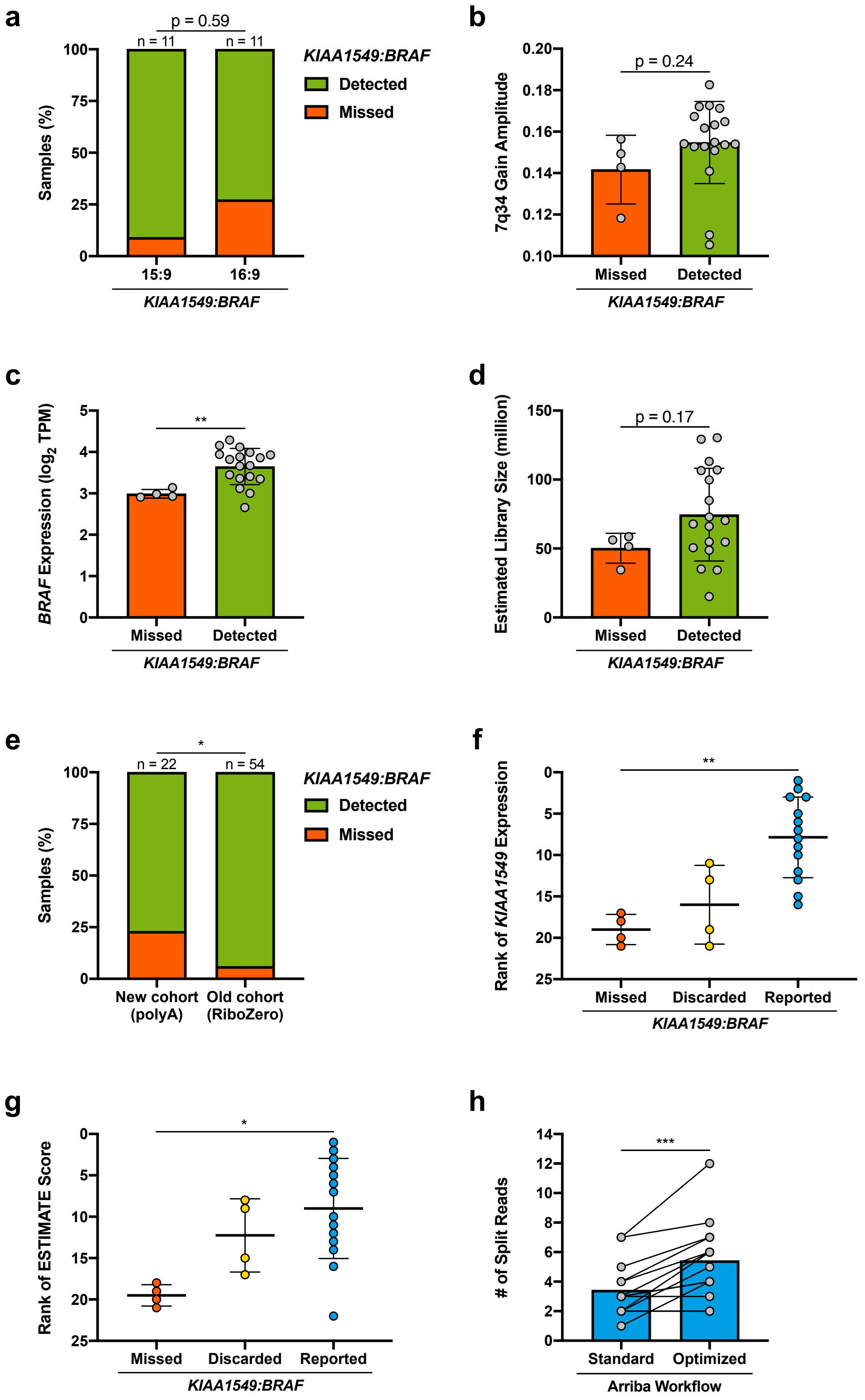

Online Resource Fig. 1 **a** Relative frequency of detected and missed *KIAA1549:BRAF* fusions in samples with known *KIAA1549:BRAF* 15:9 or 16:9 fusions. Fisher's exact test on the underlying absolute values. **b** Amplitude of the fusion-characteristic 7q34 gain extracted from methylation array-derived copy number data as an indicator of tumor cell content in samples with a missed or detected *KIAA1549:BRAF* fusion. Mean ± SD. Unpaired *t* test. **c** Expression of *BRAF* in samples with a missed or detected *KIAA1549:BRAF* fusion. Mean ± SD. Unpaired *t* test. **d** Estimated library size of samples with a missed or detected *KIAA1549:BRAF* fusion as calculated by RNA-SeQC (https://github.com/broadinstitute/rnaseqc). Mean ± SD. Unpaired *t* test. **e** Relative frequency of detected and missed *KIAA1549:BRAF* fusions in the presented cohort (polyA capture library preparation protocol) compared to an older cohort (RiboZero library preparation protocol). Fisher's exact test on the underlying absolute values. **f** Samples ranked by their *KIAA1549* expression with the highest-ranked sample having the highest expression. Mean ± SD. Kruskal-Wallis test followed by Dunn's multiple comparisons test. **g** Samples ranked by their ESTIMATE immune score with the highest-ranked sample having the lowest immune score. Mean ± SD. Kruskal-Wallis test followed by Dunn's multiple comparisons test. **h** Number of split reads identified by Arriba in the standard and optimized workflow for all samples that were initially reported. Paired *t* test. For all panels: **p* < 0.05, ***p* < 0.01, ****p* < 0.001, *****p* < 0.0001, n.s.: not significant

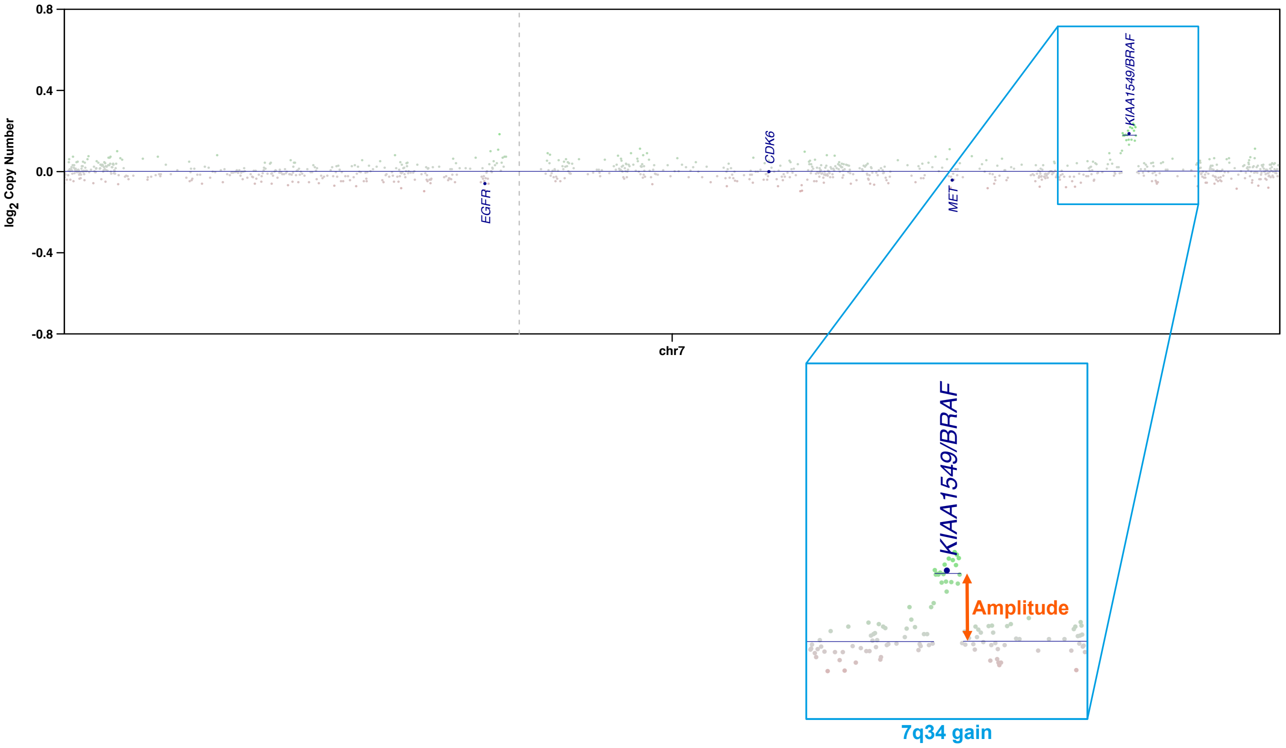

Online Resource Fig. 2 Typical copy number plot of a PA with a *KIAA1549:BRAF* fusion. The characteristic focal tandem duplication at 7q34 is magnified. The gain amplitude is measured between gain and baseline.

		Initial Sequencing		Re-sequencing				
**ID**	**Expected Fusion**	**Total Read Count**	**Arriba v1.1.0**	**Total Read Count**	**Arriba v1.1.0**	**FusionCatcher v1.20**	**grep**	**Optimized Arriba Workflow**
**1**	*KIAA1549:BRAF* 16:9	194,850,910		**676,743,058**			X	
**2**	*KIAA1549:BRAF* 16:9	207,683,590		**576,945,684**			X	X
**3**	*KIAA1549:BRAF* 15:9	163,650,066		**507,411,706**		X	X	X
**4**	*KIAA1549:BRAF* 16:9	192,301,908		**631,106,492**			X	X
**5**	*KIAA1549:BRAF* 15:9	181,693,284		**605,518,510**	discarded	X	X	X
**6**	*KIAA1549:BRAF* 15:9	213,976,502	discarded	213,976,502	discarded		X	X
**7**	*KIAA1549:BRAF* 15:9	246,750,814	discarded	246,750,814	discarded	X	X	X
**8**	*KIAA1549:BRAF* 16:9	154,956,306	discarded	154,956,306	discarded	X	X	X
**9**	*KIAA1549:BRAF* 15:9	248,395,826	X	248,395,826	X	X	X	X
**10**	*KIAA1549:BRAF* 15:9	224,107,348	X	224,107,348	X	X	X	X
**11**	*KIAA1549:BRAF* 15:9	258,025,596	X	258,025,596	X	X	X	X
**12**	*KIAA1549:BRAF* 15:9	259,592,144	X	259,592,144	X	X	X	X
**13**	*KIAA1549:BRAF* 15:9	222,007,910	X	222,007,910	X	X	X	X
**14**	*KIAA1549:BRAF* 16:9	250,453,818	X	250,453,818	X		X	X
**15**	*KIAA1549:BRAF* 16:9	221,572,204	X	221,572,204	X	X	X	X
**16**	*KIAA1549:BRAF* 16:9	212,747,662	X	212,747,662	X	X	X	X
**17**	*KIAA1549:BRAF* 16:9	233,864,126	X	233,864,126	X	X	X	X
**18**	*KIAA1549:BRAF* 15:9	201,634,092	X	201,634,092	X	X	X	X
**19**	*KIAA1549:BRAF* 16:9	198,666,870	X	198,666,870	X	X	X	X
**20**	*KIAA1549:BRAF* 16:9	179,082,272	X	179,082,272	X	X	X	X
**21**	*KIAA1549:BRAF* 15:9	208,710,688	X	208,710,688	X	X	X	X
**22**	*KIAA1549:BRAF* 16:9	187,958,056	X	187,958,056	X		X	X
			14+3		14+4	16	22	21

Online Resource Table 1 Overview of fusion detection results from RNA-Seq data of the presented cohort of fresh-frozen pediatric PA tumor samples. The higher total read counts after re-sequencing are marked in bold. X = detected
